# Developing and evaluating a customized evidence-based safety training program for healthcare staff: a quality improvement approach

**DOI:** 10.3389/fpsyt.2026.1901445

**Published:** 2026-10-27

**Authors:** Elizabeth Lin, Hugh Boyd, Emmanuel Bratsalis, Katie Hodgson, Faisal Islam, Muhammad Idrees Khan, Ninon Crestois, Sanjeev Sockalingam

**Affiliations:** 1 Education Department, Centre for Addiction and Mental Health, Toronto, ON, Canada; 2 Department of Psychiatry, University of Toronto, Toronto, ON, Canada; 3 Institute for Clinical Evaluative Sciences, Toronto, ON, Canada; 4 Sienna Senior Living, Markham, ON, Canada; 5 Department of Family Medicine, McMaster University, Hamilton, ON, Canada; 6 The Wilson Centre, Toronto, ON, Canada

**Keywords:** customized safety training, mental health care staff, quality improvement (QI), safety training evaluation, workplace violence

## Abstract

The high risk of workplace violence (WPV) for workers in mental health care settings and the potentially serious impacts, both mental and physical, have prompted many hospitals to mandate behavioral safety skills training for their staff. There are, however, some considerable challenges to delivering this type of training. First, while there are international and national/regional guidelines for mitigating WPV, the evaluation literature does not provide consistent guidance on which training methods are the most effective across a range of settings. Second, hospitals differ in terms of their objectives and their identified safety issues suggesting that a tailored approach rather than a one-size-fits-all training would be more appropriate. Finally, while there are commonly used evaluation frameworks relevant to health care, these will need to be adapted on a case-by-case basis to a tailored training approach. This article will describe models and frameworks addressing the processes involved in developing, implementing, and evaluating customized behavioral safety skills training. Preliminary findings from an actual quality improvement initiative delivered to a long-term care facility in Ontario, Canada will be presented and used as a basis to discuss where theory and actual experience converge and diverge. Successful results include the development of a replicable and theory-grounded customization process along with demonstrated short-term staff-level improvements; however, challenges remain in terms of evaluating organizational-level impact, sustainability, and resident/patient-level outcomes.

## Introduction

The decades-long attention to workplace violence (WPV), especially in healthcare, has yielded important international and national advances. The high global prevalence of WPV and ongoing need for interventions continues to be monitored (1, 2) along with evidence of WPV’s negative effects on healthcare staff (3). In 2019, the first ever international treaty recognizing the right of workers to be free from violence and harassment was introduced (4), and in 2020 workplace health and safety targets were incorporated into the United Nations “Sustainable Development Goals” (5). These initiatives have prompted the development of frameworks for violence prevention and management at both international (6, 7) and national/regional levels (8–10). These generally provide broad guidelines; however, some make very specific recommendations or are even prescriptive in terms of what outcomes should be reported or recorded – for example, the Registered Nurses Association of Ontario (10) and the U.S Occupational Safety and Health Administration (9). Use of these guidelines is supported by various national and regional mandating of safety training as a quality improvement component for hospitals and other healthcare organizations (11–13) as well as the establishment of legal and digital reporting systems in several jurisdictions for protecting victims of WPV (14).

These guidelines, while their specifics may vary, provide a fairly consistent multi-level, multi-faceted outline of the conditions and supports felt to be critical for supporting the primary objective – namely, the reduction of harassment and WPV experienced by staff. These include societal and organizational level factors (e.g., policy, legislative, environmental, leadership) as well as factors directly affecting staff (e.g., education, de-escalation training, reporting and protection protocols). The difficulties for organizations wishing to prevent or decrease WPV are at least two-fold. First, multi-faceted solutions are quite complex to design, implement, and evaluate (see for example, 15). Second, evaluation of even straightforward solutions is challenging. Indeed, in a scoping review of 77 studies from over 50 countries across six continents, Fiorino et al., only found 11 that reported evaluations of their interventions (1).

Evaluations of WPV strategies, when they are done, yield promising but inconsistent results. Synthetic literature reviews of staff safety training evaluations almost universally report that the impacts of training on outcomes such as staff confidence or WPV decreases are not consistently demonstrated (16–21). In terms of other kinds of WPV-management strategies, an evidence review of individual-, organizational-, and community-level interventions concludes that they are inadequate when examined alone (22). On the other hand, a systematic review which included five studies of multi-component programs found positive impacts on rates of WPV (21). The variation among the five studies precludes determining the effectiveness of specific intervention combinations although the authors do note the inclusion of stakeholders as an important ingredient. Recommendations for addressing the evidence gaps identified in the literature include more rigorous study designs, more consistent definitions of interventions and WPV across studies, or testing multimodal or multi-system level interventions with the assumption that such changes will improve detection of training impact. Our own experience in attempting a more rigorous design suggests that this does not in itself correct the lack of consistent training results (23). Further, both rigorously designed and multi-component evaluations, despite their potential scientific benefits, may be impractical for many organizations wishing to evaluate their management programs in an efficient and timely manner to address their WPV gaps.

We argue that a different, but potentially valuable alternative from a pragmatic perspective is an adaptive customization, implementation, and evaluation of an evidence-based safety skills training. Our objective is to describe such an approach (the Trauma-Informed De-escalation Education for Safety and Protection -- TIDES™) along with preliminary evaluation results and a description of the processes used to incorporate some of the contextual and multi-level factors identified in the guidelines and the literature (e.g., organizational policy, leadership, environment, and staff/stakeholder involvement -- 7, 21). In addition, we will describe factors specific to the training site -- including the impact of the COVID-19 pandemic, the associated escalation in challenging resident behaviors, leadership changes, and space/time/scheduling constraints and also discuss lessons learned and implications. Our hope is that publication of our approach may contribute to a framework to advance this type of customized work and future data collection and analysis.

Following the Canadian policy governing ethical research (24) that quality improvement initiatives need ethics oversight only when results are to be published, our institutional review board was consulted on both the plan for this publication as well as the supporting curriculum and evaluation. They have concluded that this constituted a quality improvement initiative with no research ethics review required.

The SQUIRE reporting checklist (25) was used in editing this document (see **Appendix A**).

## Context

The site for the safety skills training was an Ontario-based long-term care facility (LTCF) that is part of a national Canadian organization offering retirement, long-term, and continuing care services. The requested training was for the long-term, continuing care component which provides care for approximately 160 residents annually. Roughly half of the clientele are older adults; half also have chronic mental illness but are not necessarily older adults. The LTCF consists of five units, including residents with more behavioral needs and employs 330 resident-facing staff and providers. These include physicians, nurses, recreational therapists, personal and general support workers, and some general support staff who provide 24/7 nursing and personal care, onsite medical care, palliative and end-of-life support as well as dining, recreation, and rehabilitation services. These are supported by the LTCF’s executive and administrative leadership as well as specialized services such as behavioral supports trainers.

The specific impetus for the LTCF’s request was escalating behavior by a resident who had initially been housed in CAMH before transition in care. More broadly, however, the transition and interruptions in support due to the COVID-19 pandemic resulted in increased calls to the police for dealing with increasing numbers of LFTC residents exhibiting escalated behavior. This resulted in the facility’s leadership contacting the TIDES™ program leadership in an attempt to address this problem.

## The intervention

TIDES™ was developed by the Centre for Addiction and Mental Health (CAMH) in 2018. Grounded in evidence-based continuing professional development design and implementation (26–28) and incorporating health equity in its design and delivery (29), it takes a comprehensive approach that spans multiple phases of risk management from prevention and early identification to collaborative de-escalation, team-based responses, and the use of physical skills to facilitate restraint when necessary.

The curriculum is guided by trauma-informed and patient-centred care principles and, in keeping with adult learning approaches, combines didactic teaching, simulation, and practice-based learning. Evaluation is integrated throughout using a variety of trainee, observer-rated, leadership, and organizational data sources. Training therefore emphasizes a coordinated response across stakeholders and the application of skills within real-world care environments. TIDES™ training has been mandatory for CAMH staff for 7 years. TIDES™ trainers are allied healthcare professionals with both clinical and safety training experience who undergo on-boarding and follow-up mentoring to acquaint them with the curriculum content and process. TIDES™ has been implemented externally using a customization approach for a range of clinical settings, including community-based, hospital-based, and other healthcare organization contexts, of which this report is an example.

### Adapting TIDES™

The proposed curriculum was customized, in consultation with the LTCF, based on existing TIDES™ expertise and instructional materials. The training design and implementation were informed by the Analysis, Design, Develop, Implement, and Evaluate (ADDIE) approach (30) and the needs-assessment based model described by Kaufman and colleagues (31). However, the processes and recommended steps from these two frameworks were streamlined to balance methodological rigor with urgent need. The LTCF workers were experiencing distress and serious WPV challenges, and the leadership felt that rapid implementation was needed.

The ADDIE approach, introduced in the 1970s (32), is one of the most widely used and emulated methodologies for instructional design. Its goal is to systematically guide the creation of ‘field-effective and efficient instruction’ to prepare workers to meet real-world performance requirements (30, p.434). The focus is thus on imparting standardized skills and capabilities needed for performance in a structured work environment.

Kaufman et al. (31)’s focus is on designing program interventions. Building on a tradition of grounding program development in needs assessment (e.g., 33), they describe a conceptually and operationally complex framework that moves from ascertaining and defining needs to choosing, implementing, and evaluating potential program interventions.

While these two models originated for different purposes, they outline similar processes (30, 31, 33, 34). Both begin with analysis or needs assessment to identify and, particularly, to prioritize needs. This information then supports the design, development, and implementation of the program and sets the stage for the evaluation activities.

## Analysis/needs assessment processes

The LTCF and the TIDES™ team organized staff focus groups from different work shifts and departments/floors to elicit their concerns and perceptions on possible solutions, including which priorities should incorporated into a short (one-day) training. The LTCF also provided detailed reports on serious incidents of escalated behavior (Code Whites) that had led to police contact (911 calls). The TIDES™ team summarized themes from the focus groups and Code White material and presented and discussed them with both the LTCF leadership and staff. These discussions resulted in the creation of five objectives that were both priorities to the facility and could be feasibly addressed in the one-day training. The TIDES™ team also proposed additional topics based on their experience with what previous organizations and facilities had found useful.

## Study design

As shown in **Table 1**, these objectives and additional topics (Column 1) were evaluated using a combination of pre-post and longitudinal designs. For the staff survey, a one-group, multiple time point design was used (pre-, post-, follow-up without comparison group -- 35). The director survey design was a simple pre-post while the administrative data assessment was a monthly longitudinal assessment for the 12 months before and the 12 months after the training. Surveys included a few open-ended questions (e.g., how could the training session be improved)?, but these responses were not integrated with or used to interpret results from the close-ended survey questions. All evaluation tools were developed following standard ethical practices including provision of informed consent to participants prior to starting the surveys, anonymous collection of responses, and data processing by a trained, blinded analyst. Survey items were selected from widely accepted and commonly used measures (e.g., confidence, perceived competence) and modified to fit the agency’s identified priorities.

**Table 1:** Evaluation plan.

Objective	Staff survey observer-rated skills			Director/administrator survey		Administrative data
	Baseline	Post-training	1-month Follow-up	Baseline	1-month Follow-up	Monthly: 1-year pre to 1-year post-training
Kirkpatrick training evaluation level	1 & 2 (Reaction, Learning) 3 (Behavior)			4 (Results)		
LTCF (long-term care facility)-identified objectives						
Reducing Responsive Behaviors	X	--	X	X	X	X
Increasing Resident Cooperation	X	--	X	--	--	X
Decreasing Staff and Resident Injuries	X	X	X	--	--	X
Reducing the Frequency of Code Whites	X	X	X	--	--	X
Minimizing the Need for Police Intervention	X	X	X	--	--	X
Additional topics (TIDES™ Trainer-generated)						
Perceptions of safety, Self-efficacy, confidence	X	X	X	--	--	--
Effectiveness of training	X	X	X	X	X	--
Areas for improvement	--	X	X	X	X	--

### Training design, development, and implementation processes

The prioritized needs and additional topics then drove the design and development of the one-day training. Existing TIDES™ training materials, methods, and evaluation questions were examined for how well they captured or could be modified to address LTCF objectives within its practical limitations. In addition, new tools that might provide more detail to the evaluation findings were added (see Evaluation section below). The key characteristic of this process is that the curriculum content was directly linked to both the training objectives and the evaluation questions so that a coherent, easily interpretable set of findings could be produced.

The teaching methods chosen also drew on the foundational TIDES™ curricula, combining lectures describing trauma-informed care, behavioral skills and experiential training for physical protection skills, simulation-practice, and embedded time for group reflection. Content utilized case examples, for example, code white scenarios, from the LTCF to support application to real work clinical settings and translation of skills into practice.

The outline and learning objectives for the final one-day training are shown in **Appendix B**.

### Evaluation processes

Although evaluation is listed as the last step in both models (e.g., the **e** in ADDIE), it is intended to be conducted throughout the entire instructional or program design. Formative evaluations are used to assess the analysis, design, and development processes in terms of how well they reflect the identified objectives. Later, operational evaluations are used routinely to test the curriculum or program implementation and to prompt adjustments in how the training is delivered. Finally, summative evaluations are used to assess how well the training or program outcomes meet the desired objectives.

Our choice of evaluation measures was guided by Kirkpatrick’s framework (36, 37) because it seemed better suited than other widely used frameworks (e.g., Moore’s (38)) for the LTCF’s needs. Originally developed as a tool for businesses to improve training programs, it is comprised of four levels. The specific feature of Kirkpatrick which was felt most relevant to our evaluation was its prioritizing of organizational objectives (the fourth level) as the central driver for the evaluation (39):

Level 1: Reaction: trainee sense of how relevant and engagement the training is
Level 2: Learning: trainee acquisition of knowledge, skills, and attitudes
Level 3: Behavior: trainee use of that learning on the job
Level 4: Results: changes in the outcomes targeted by the organization

The evaluation items drawn or modified from existing TIDES™ materials covered the types of injuries staff sustained from encounters with aggressive residents, their confidence in dealing with residents with escalating behavior and with incidents requiring calls to the police, their assessment of how effective they felt the training was, plus open-ended questions about the training and areas for improvement. These items comprised the staff survey which was designed to be administered just before training (baseline), immediately after (post-training), and at one-month follow-up. We also proposed observer-rated assessments for the physical safety component to evaluate skill acquisition and retention, to be administered at those same timepoints.

Other information sources were added to allow a more complete evaluation related to the LTCF’s priority objectives and additional topics. To gain a more in-depth understanding about the impact training might have on staff, we supplemented the existing TIDES™ bespoke questions about trainee confidence with two validated, published scales measuring self-efficacy (40) and mental well-being (41). To ascertain the organizational perspective, we designed a short survey for directors and administrators asking their impressions of how well the LTCF had been able to handle behavioral emergencies in the past month. We also included analyses of routinely collected administrative data. These include the frequency of abuse events, the subset that are critical incidents, calls to the police (911), transfers to the emergency department, and staff injuries. These are collected on an ongoing basis by the administrative and management personnel and are independent from the self-reported information by the staff collected for the curriculum evaluation.

The director/administrator survey was designed to be administered at baseline and one month after training, while the administrative data analysis would cover monthly LTCF statistics for the year before and the year after training.

In tailoring the existing TIDES™ evaluation questions to the LTCF-identified objectives, we considered all four Kirkpatrick levels as well as the potential data sources that might provide the needed information (see **Table 1**). Levels 1 and 2 are addressed by the staff surveys which query trainees’ impressions of the value and effectiveness of the curriculum and Level 4 by results from the planned analysis of the LTCF’s administrative data. For Level 3, we planned a proxy measure of behavior – an observer-rated assessment of how well the trainee learned or retained the physical safety skills being taught. However, this was deemed impracticable given the LTCF’s limited time and space resources. In addition, changes in the LTCF leadership just when the director/administrator surveys were to start led to difficulties having them completed. Consequently, both the observer-rated assessments and the director/administrator surveys have been dropped from the evaluation plan. (Final survey questions available from Corresponding Author upon request).

## Results

The results reported here are from the baseline and post-training surveys for the 118 trainees who have completed the safety training to date. Non-response ranged from 0 to 12 percent, depending on the questionnaire item. Training is continuing as well as data-collection from the 1-month follow-up surveys and administrative data.

**Table 2** and **Figure 1** show selected staff responses related to the five LTCF-identified objectives. Indicators for two objectives (Reducing Responsive Behaviors, Increasing Resident Cooperation) cannot yet be calculated because they depend on the 1-month follow-up data. However, for three objectives (Decreasing Staff and Resident Injuries, Reducing the Frequency of Code Whites, and Minimizing the Need for Police Intervention are available), the results show that from the staff perspective, the training has had a positive impact. For all three objectives, staff average scores of effectiveness rose from 3.8 to 4.2-4.5, on a five-point scale.

**Table 2:** Selected evaluation results (staff surveys) for LTCF-identified objectives (see **Appendix C** for numerical and statistical details).

LTCF objective	Baseline	Post-training
Reducing Responsive Behaviors	In the past month, how safe have you felt at work? Somewhat not safe to not safe at all: 22% Somewhat safe to completely safe: 58%	To be collected at 1-month follow-up
Increasing Resident Cooperation	How often do residents cooperate with mandatory care procedures without resistance? Always/often: 31% Rarely/never: 6%	

**Figure 1:**
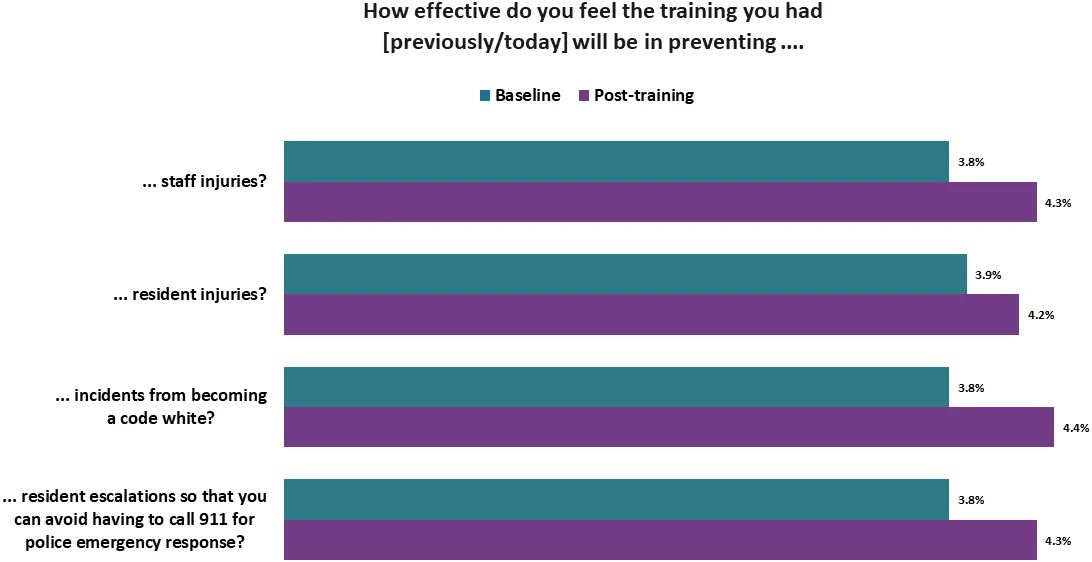
Training effectiveness in preventing injuries and 911 calls.

**Figures 2**–**5** show selected staff responses related to the trainer-identified additional topics. Again, from the staff perspective, the results suggest that the training had a positive impact on self-efficacy with a baseline mean score of 53.4 (s.d. 12.7) compared to a post-training mean of 72.3 (s.d. 31.8), how safe staff felt around aggressive residents (somewhat to very safe: 68% vs 86%), and how effective they felt the TIDES™ training was (effective/very effective: 65% vs 83%).

**Figure 2:**
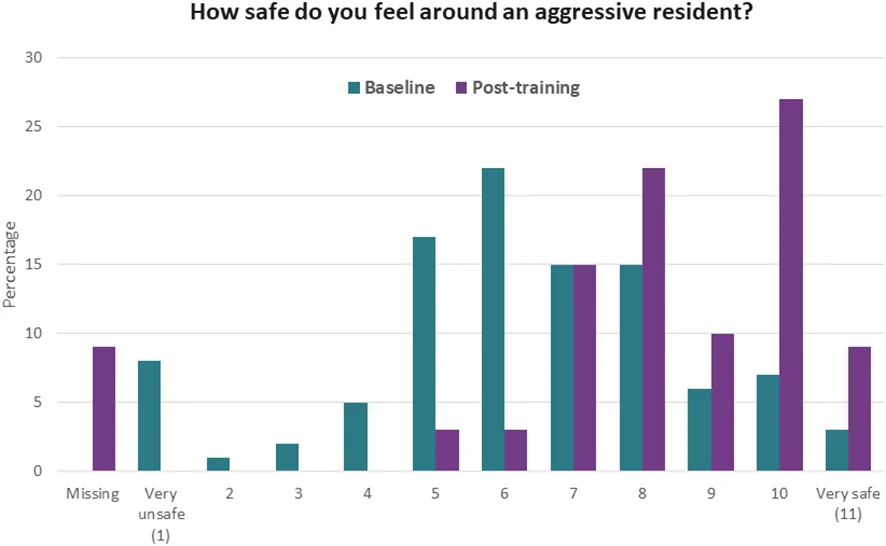
Perceptions of safety around an aggressive resident.

**Figure 3:**
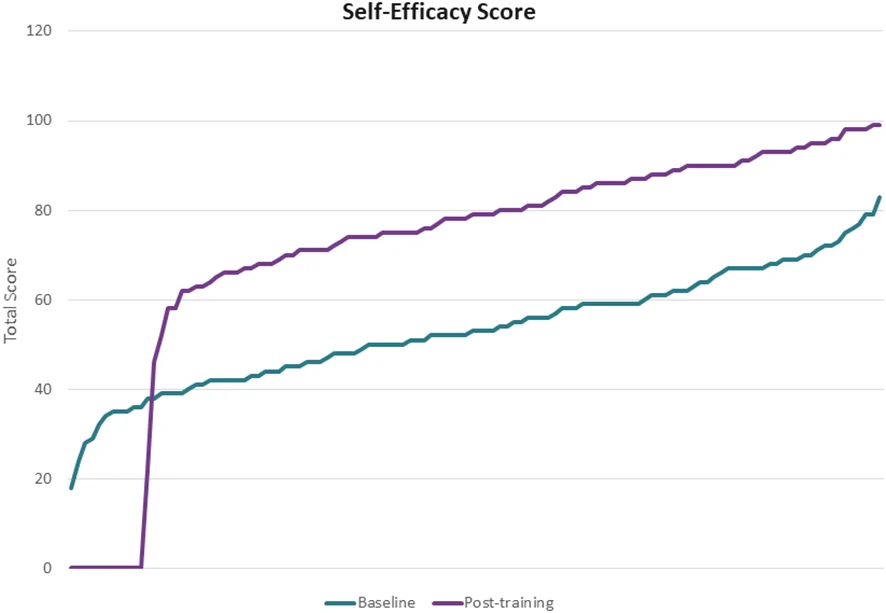
Self-efficacy score (adapted from Thackery, (40)).

**Figure 4:**
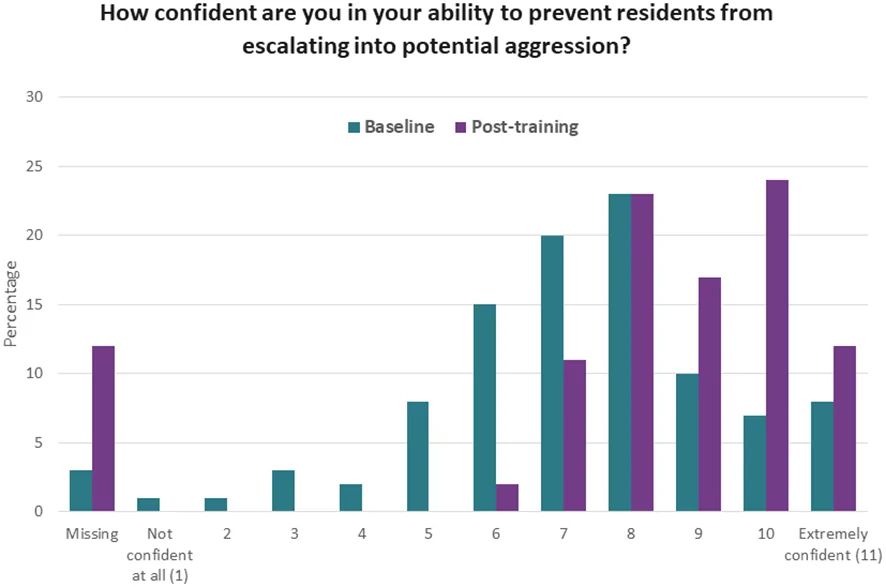
Confidence in preventing escalation to potential aggression.

**Figure 5:**
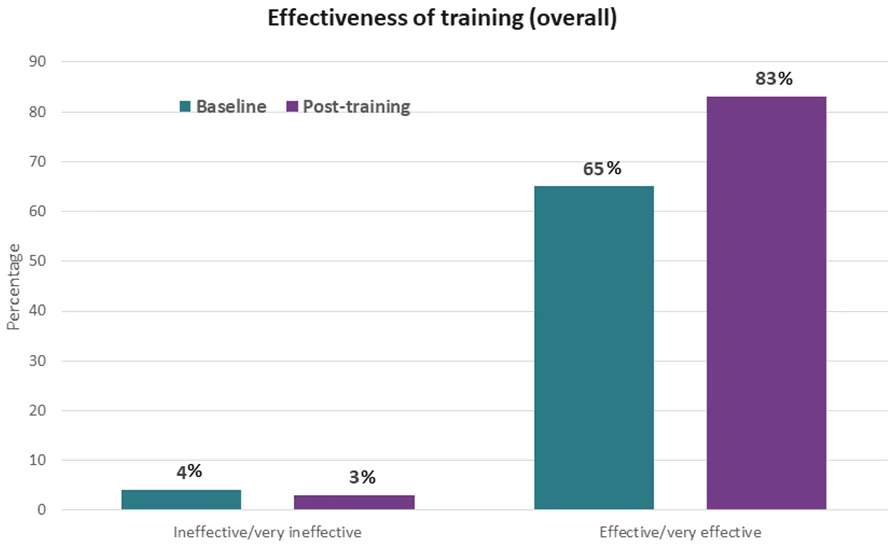
Effectiveness of training (overall).

Trainees were also asked an open-ended question about whether there were aspects of the training that could be improved. Their responses were thematically analyzed, and **Table 3** shows their responses for the three most commonly endorsed themes.

**Table 3:** Staff-identified areas for training improvement.

Was there any part of the training that you found less relevant or could be improved?	
Theme (number of comments)	Selected quotes
Culture of safety when working with the older adult population (n=11)	*“It was fine according to my opinion some areas could be improved by giving more examples.”* *“BSO* [Behavior Supports Ontario] *should follow up with the clients”* *“Monthly of weekly team meetings to improve the quality of care with patient centered approaches as not all team members are aware of the resident’s behaviors/triggers/how to de-escalate when things are not well.”*
Team response to escalated situations and Code White response (n=9)	*“Do another scenario where a patient is in the wheelchair and is having behaviors.”* *“Team response should be taken as single care giver can find it hard cause an aggressive resident can harm any way.”* *“Identified on who the leader is during a code white.”*
Self-protection skills at the bedside (n=8)	*“To ask some questions regarding some situations that happen bedside.”* *“at bedside to wake the resident feel safe less aggressive it should be trained to all staff at LTCF.”* *“Would be better if we had a bed included.”*

## Discussion

The preliminary short-term evaluation results indicate that the staff perceive the training to be useful and effective. Managers noted the shared language and approach to understanding trauma, triggers, and behavioral escalation helped with multi-disciplinary engagement that integrated with their existing long-term care practices. Further, there are some organizational responses that support this positive impression. Anecdotal reports indicate that calls to the police have stopped since the start of training demonstrating early impact. In addition, the LTCF leadership requested a three-day Train-the-Trainer (TTT) curriculum signaling their desire to expand their in-house expertise and capacity for on-going safety skills education. Our sense is that this additional request stemmed both from the staff’s initial positive reactions as well as the rapport, engagement, and subsequent buy-in established by the continual inclusion of the LTCF’s leadership and staff throughout the design, delivery, and evaluation process. Initial evaluation of the TTT indicates highly positive impact as reported by the trainees including marked increases in knowledge, de-escalation and self-protection skills, and confidence in training their fellow staff members. The LTCF’s parent organization is currently discussing scale and spread of this TTT with the plan to first build on the processes described here to customize training for two other regional sites and then possibly spread nationally depending on the regional results. Further verification of whether these positive impressions will persist over time await successful collection of follow-up surveys and administrative data analysis as well as the regional TTT evaluations.

Our experience has shown, not unexpectedly, that while ADDIE, the needs-based approach described by Kaufman and colleagues (31), and Kirkpatrick’s evaluation framework (39) are extremely valuable in providing operational and conceptual guidelines, adhering to them faithfully is challenging. For example, in Kaufman, et al.’s approach, needs are identified using a logic model to determine discrepancies between desired and observed outcomes and verified using ‘valid, objective data.’ (31, p.8). They also provide a classification table linking particular types of identified needs to recommended actions (31) while Peterson details a lengthy process by which instructional courses are designed, developed, and implemented (34).

However, the LTCF’s need for rapid implementation generated a more pragmatic adaptation. For example, the leadership’s concern about increased calls to police was accepted as demonstrating both legitimate need and as well as signaling gaps in staff knowledge and skills without direct assessment via ‘valid, objective data’ (31, p.18) or analysis of any logic model or theory of change (31). Priority setting, while it did involve the organization’s perspective, was done in a semi-structured rather than formal manner (iterative discussions with leadership and staff). At the same time, the approach was not exclusively deficit-focused. Consistent with Altschuld and Kumar’s (42) strengths-informed perspective, the process also recognized existing organizational responsiveness, leadership engagement, and staff willingness to adapt as important assets that could support implementation.

In addition, we anticipate future challenges as the training continues. Changes in personnel and demanding staff schedules may continue to compromise the collection of follow-up data making evaluation of the retention of training impact difficult and requiring iterative evaluation methods that may be beyond the capacity of the LTCF. Indeed, as of the current date, we have learned that the follow-up staff assessment survey has had an extremely high non-response rate (nearly 90%), which will necessitate an investigation into the root causes as well as a discussion about how to improve implementation. Further, while the administrative data is routinely collected, finding the needed staff and time resources may hinder generating the planned data reports.

This specific evaluation provides a rationale for a customized approach to safety skills training that is timely, adaptive and responsive to an organization’s needs. The LTCF’s acute needs, urgent timelines and resource limitations necessitated the need to be nimble and for the TIDES™ team to engage and co-produce the best solution within those limitations. Within these constraints, the direct alignment of the information collected to the LTCF’s priorities of decreasing injuries, Code Whites, and calls to the police provided meaningful and practical indicators. This approach is aligned with past reviews indicating the need for multicomponent interventions to improve skills in managing and preventing WPV with ongoing monitoring to adjust to needs (17).

The need for the LTCF to be resident- and care-focused also meant that design and evaluation elements such as ongoing formative and operational evaluations, objective skills assessment, and administrative data verification of organization-wide results likely have less value than staff narratives and anecdotal patient/client reports for the LTFC staff and leadership. Thus, elements and processes highlighted as important in the literature may not have the same priority from an administrative and service delivery viewpoint. Continuous communication between the TIDES™ team and the LTCF organization’s leadership was necessary for the TIDES™ team to outline the strengths and limitations of the results so that the organization’s leadership could make informed decisions on how to proceed further. In addition, we hope to leverage the jointly developed rapport and collaboration with the agency to expand future evaluations. Candidates for addition include assessing the perspectives of the agency’s residents; the staff’s perception and experience of the protocols and administrative response to reporting incidents; or even environmental supports and barriers to preventing WPV.

The critical question is what contribution do such customized approaches make to the practical utility versus the body of scientific knowledge on safety training. Existing guidelines and frameworks recommend what the originators consider to be the full set of levels, steps, and components necessary for a complete and sound product. The implication is that our evaluation results of the TIDES™ customized program only show a partial and limited picture. While our customization process attempted to incorporate or at least acknowledge the importance of leadership and stakeholder support, the LTCF’s pragmatic needs precluded a thorough assessment of the recommended factors (7) or the socio-ecological levels suggested by some current frameworks (43). Even from a narrower perspective of focusing on the training itself, our evaluation is incomplete. Three of the more consequential gaps are the lack of the director/administrator survey and the observer ratings of skill performance as well as the high non-response rate for the follow-up staff survey.

As noted above, the director/administrator survey was dropped because of leadership personnel changes during the start of training. Even if the new leadership completes the survey, this will only provide retrospective or anecdotal information about training impact since a true pre-training evaluation is now impossible. Thus, if our efforts to obtain the LTFC administrative data are not successful, there will be no organization-level information to complement the staff-level results for evaluating the training.

Regarding the observer-rated skills, we previously found that physical skills safety training had a positive post-training impact on both staff confidence and observable skills acquisition. However, at one-month follow-up, while self-reported confidence levels remained high, observer-rated skill retention levels dropped by as much as 25 to 33 percent, depending on the specific skill (23). Removal of the observer-rated skill assessments means that there is no reliable way to assess LFTC staff skill retention since the self-reported confidence and self-efficacy measures are not adequate proxies for objectively-rated skill level. A potential solution being discussed with the LFTC parent organization would be to include abbreviated pre-, post-, and follow-up videotaped sessions in the scale-spread TTT evaluations. The plan will be to limit the videotapes and associated blind ratings to two key protection skills (instead of the 11 assessed in our previous study (23) as a way to reduce resource demands and burden.

The low response rate for the follow-up survey, given our previous work, is particularly concerning since there is currently no way to document even self-reported skill retention. Analysis of the administrative data, if successfully implemented, might provide results where skill retention or lack thereof could be inferred or speculated about. The abbreviated TTT observer-rated assessments might also provide information about the skill retention of the trainers, but this would only provide suggestive evidence about the staff’s skill retention.

In sum, the contributions of our approach include a customization process that is theory-grounded, replicable, and demonstrates short-term staff level outcomes that align with organizational priorities. These are characteristics that support the practical utility of our results, at least in the short term. However, we have not, to date, been able to provide evidence about impact at the organizational level, sustainability of the training, nor the perspective of the LTFC’s residents. These are concerning limitations in terms of both longer-term practical utility as well as the completeness of any contributions made to the scientific literature.

Solutions addressing these limitations include continual collaboration with agency partners to set appropriate expectations of the customized program and, ideally, on-going monitoring and exploration of available data and evaluation possibilities to mutually identify further training and evaluation improvement opportunities.

Perhaps another solution would be to contribute to and rely on the developing and sophisticated methods used by synthetic literature reviews and meta-analyses to find patterns across heterogeneous studies. For example, evaluation results could be reported in a way conducive to metanalysis or similar complex methods (see, for example, 16). Providing means and standard deviations for all groups and conditions (for continuous measures) or reporting raw frequencies and denominators (for categorical measures) or at least indicating that they are readily available might assist in this (see **Supplementary Material** for the numerical and statistical information related to our **Tables 2**, **3**). As another example, Provost, et al. (44) in a realist review of WPV prevention measures identified important contributors to three key outcomes: decreasing WPV incidence, decreasing harm to workplace staff, and increasing the reporting of WPV. The particular value of their analysis was that the contributors were not single factors but configurations of contexts, mechanisms, and outcomes instead – an analysis more suited to the complex and interconnected patterns outlined in WPV prevention and reduction frameworks.

## Conclusion

Customized training can be done relatively quickly and, in a manner, closely responsive to organizational or facility needs. Based on our experience, the key ingredient was the early and continual engagement and inclusion of the LTCF at multiple levels (administration, management, staff). Indeed, anecdotal reporting from the LTCF indicated that the staff were highly appreciative that their concerns were listened to and addressed in a way that was both responsive and useful to their practice. While staff feelings of inclusion were not identified as a primary objective, our sense is that this may be one of the more valuable and consequential outcomes of the customization process.

Other important ingredients were the ability of the LTCF and the TIDES™ team to arrive at mutually agreed upon objectives and the prior experience of the TIDES™ team in creating and implementing the final one-day training.

Conceptual and guiding frameworks also make an important contribution despite their being superseded at times by pragmatic concerns. Their value lies in providing a systematically organized road map comprised of linked components which guides intervention design. They also highlight limitations and gaps and thus contribute to more tailored, actionable decision-making by the organization or facility.

As noted earlier, pragmatic barriers may continue to limit the content and completeness of the evaluation findings – specifically in our case, missing objectively assessed skill retention, other longer-term impacts of the training, and the organizational outcomes. Some of these are not unexpected. For example, Level 4 of the Kirkpatrick framework (i.e., the organizational-level results) is the critical driver of determining what outcomes to assess (39); yet it is not uncommon that these data are not consistently reported (45). The challenge is how to interpret and mitigate such partial results from both real-world actionability and scientific rigorousness lenses. We have suggested a few solutions (e.g., discussing limitations of the results with the LTFC in terms of their next steps, building on existing standardized scales, continuing to explore ways to make the evaluations more comprehensive, reporting findings so they are usable for synthetic literature reviews) with the expectation that others encountering similar issues will also provide alternatives.

## Data Availability

This paper is a secondary analysis of quality improvement/evaluation data and as per agreement, is only available publicly in aggregated, de-identified form for approved reasons. Requests to access these datasets should be directed to Elizabeth Lin, elizabethbetty.lin@camh.ca.
