# Supplementary material for: Developing and evaluating a customized evidence-based safety training program for healthcare staff: a quality improvement approach
Source: Front Psychiatry. 2026 Oct 27;17:1901445. doi: 10.3389/fpsyt.2026.1901445 (PMC13748346; doi:10.3389/fpsyt.2026.1901445)
Supplement: Supplementary file 1 [file SupplementaryFile1.docx]

APPENDIX A: SQUIRES Reporting Checklist (Ogrinc et al. 2025)

The SQUIRE reporting checklist

|  | Item Description | Location (or reason for not reporting) |
| --- | --- | --- |
| **Title and Abstract** |  |  |
| [1. Title](https:/resources.equator-network.org/reporting-guidelines/squire/items/title.html?utm_source=squire&utm_medium=checklist&utm_campaign=SQUIRE_2_0_2016_v1_1) | Indicate that the manuscript concerns an initiative to improve healthcare (broadly defined to include the quality, safety, effectiveness, patient-centredness, timeliness, cost, efficiency and equity of healthcare). | Title |
| [2. Abstract](https:/resources.equator-network.org/reporting-guidelines/squire/items/abstract.html?utm_source=squire&utm_medium=checklist&utm_campaign=SQUIRE_2_0_2016_v1_1) | 2a. Provide adequate information to aid in searching and indexing.  2b. Summarise all key information from various sections of the text using the abstract format of the intended publication or a structured summary such as: background, local problem, methods, interventions, results, conclusions. | Abstract |
| **Introduction** |  |  |
| [3 & 4. Problem description & Available Knowledge](https:/resources.equator-network.org/reporting-guidelines/squire/items/problem-description-available-knowledge.html?utm_source=squire&utm_medium=checklist&utm_campaign=SQUIRE_2_0_2016_v1_1) | 3. Nature and significance of the local problem. 4. Summary of what is currently known about the problem, including relevant previous studies. | Introduction, paragraphs 1-3 |
| [5. Rationale](https:/resources.equator-network.org/reporting-guidelines/squire/items/rationale.html?utm_source=squire&utm_medium=checklist&utm_campaign=SQUIRE_2_0_2016_v1_1) | Informal or formal frameworks, models, concepts, and/or theories used to explain the problem, any reasons or assumptions that were used to develop the intervention(s), and reasons why the intervention(s) was expected to work. | The Intervention and Adapting TIDES^tm^ sections |
| [6. Specific aims](https:/resources.equator-network.org/reporting-guidelines/squire/items/specific-aims.html?utm_source=squire&utm_medium=checklist&utm_campaign=SQUIRE_2_0_2016_v1_1) | Purpose of the project and of this report. | Introduction, paragraph 4 |
| **Methods** |  |  |
| [7. Context](https:/resources.equator-network.org/reporting-guidelines/squire/items/context.html?utm_source=squire&utm_medium=checklist&utm_campaign=SQUIRE_2_0_2016_v1_1) | Contextual elements considered important at the outset of introducing the intervention(s). | Context section |
| [8. Intervention(s)](https:/resources.equator-network.org/reporting-guidelines/squire/items/interventions.html?utm_source=squire&utm_medium=checklist&utm_campaign=SQUIRE_2_0_2016_v1_1) | A. Description of the intervention(s) in sufficient detail that others could reproduce it B. Specifics of the team involved in the work. | The Intervention and Adapting TIDES^tm^ sections  Intervention section, paragraph 2 |
| [9. Study of the Intervention(s)](https:/resources.equator-network.org/reporting-guidelines/squire/items/study-of-the-interventions.html?utm_source=squire&utm_medium=checklist&utm_campaign=SQUIRE_2_0_2016_v1_1) | A. Approach chosen for assessing the impact of the intervention(s) B. Approach used to establish whether the observed outcomes were due to the intervention(s). | A. Evaluation processes section  B. Study design (no comparison or control group) did not allow rigorous determination of causation) |
| [10. Measures](https:/resources.equator-network.org/reporting-guidelines/squire/items/measures.html?utm_source=squire&utm_medium=checklist&utm_campaign=SQUIRE_2_0_2016_v1_1) | A. Measures chosen for studying processes and outcomes of the intervention(s), including rationale for choosing them, their operational definitions, and their validity and reliability B. Description of the approach to the ongoing assessment of contextual elements that contributed to t… | A. Table 1 (Evaluation plan)  Specific measures in Tables 2 and 3, Figures 2-5  B. Not applicable. Contextual elements incorporated into the adaptation and implementation process and thus not independently measured. |
| [11. Analysis](https:/resources.equator-network.org/reporting-guidelines/squire/items/analysis.html?utm_source=squire&utm_medium=checklist&utm_campaign=SQUIRE_2_0_2016_v1_1) | A. Qualitative and quantitative methods used to draw inferences from the data B. Methods for understanding variation within the data, including the effects of time as a variable. | Not applicable. Analyses applied only to needs assessment to help determine content of customized training.  Only descriptive statistics used for Results with no tests of significance. |
| [12. Ethical considerations](https:/resources.equator-network.org/reporting-guidelines/squire/items/ethical-considerations.html?utm_source=squire&utm_medium=checklist&utm_campaign=SQUIRE_2_0_2016_v1_1) | Ethical aspects of implementing and studying the intervention(s) and how they were addressed, including, but not limited to, formal ethics review and potential conflict(s) of interest. | Introduction, paragraph 6 |
| **Results** |  |  |
| [13 a & b. Evolution of the intervention and details of process measures](https:/resources.equator-network.org/reporting-guidelines/squire/items/intervention-evolution-process-measures.html?utm_source=squire&utm_medium=checklist&utm_campaign=SQUIRE_2_0_2016_v1_1) | 13a. Initial steps of the intervention(s) and their evolution over time (e.g., time-line diagram, flow chart, or table), including modifications made to the intervention during the project.  13b. Details of the process measures and outcome. | Table 1 (Evaluation plan)  Specific measures in Tables 2 and 3, Figures 2-5  Appendix B (outline and learning objectives for final training) |
| [13 c, d & e Contextual elements and unexpected consequences](https:/resources.equator-network.org/reporting-guidelines/squire/items/context-unexpected-consequences.html?utm_source=squire&utm_medium=checklist&utm_campaign=SQUIRE_2_0_2016_v1_1) | 13c. Contextual elements that interacted with the interventions  13d. Observed associations between outcomes, interventions and relevant contextual factors  13e. Unintended consequences such as benefits, harms, unexpected results, problems or failures associated with the intervention(s) | Context section.  Associations inferred (see Discussion) but not actually tested.  Discussion, paragraphs 2-4 |
| [13 e. Missing data](https:/resources.equator-network.org/reporting-guidelines/squire/items/missing-data.html?utm_source=squire&utm_medium=checklist&utm_campaign=SQUIRE_2_0_2016_v1_1) | 13e. Details about missing data. | Not applicable (information only provided from respondents who completed both the pre- and post- training surveys; relatively low non-response rates – under 15% depending on question; confidentiality and privacy procedures prevent individual analyses of non-respondents) |
| **Discussion** |  |  |
| [14. Summary](https:/resources.equator-network.org/reporting-guidelines/squire/items/summary.html?utm_source=squire&utm_medium=checklist&utm_campaign=SQUIRE_2_0_2016_v1_1) | 14a. Key findings, including relevance to the rationale and specific aims  14b. Particular strengths of the project | Positive results related to the stated priorities and objectives (Tables 1-3, Figures 2-5)  Establishment of positive collaboration between training team and client agency. Continuing interest by client agency to expand in-house capacity to deliver safety training |
| [15. Interpretation](https:/resources.equator-network.org/reporting-guidelines/squire/items/interpretation.html?utm_source=squire&utm_medium=checklist&utm_campaign=SQUIRE_2_0_2016_v1_1) | 15a. Nature of the association between the intervention(s) and the outcomes  15b. Comparison of results with findings from other publications  15c. Impact of the project on people and systems  15d. Reasons for any differences between observed and anticipated outcomes, including the influence of context  15e. Costs and strategic trade-offs, including opportunity costs | 15a. Positive self-reported results (both from surveys and organizational anecdotes) of short term impacts of training  15b. No directly comparable results in terms of specific measures.  15c. Discussion section  15d. Not applicable  15e. Not applicable – not identified by client or team as priorities |
| [16. Limitations](https:/resources.equator-network.org/reporting-guidelines/squire/items/limitations.html?utm_source=squire&utm_medium=checklist&utm_campaign=SQUIRE_2_0_2016_v1_1) | 16a. Limits to the generalisability of the work.  16b. Factors that might have limited internal validity such as confounding, bias, or imprecision in the design, methods, measurement, or analysis.  16c. Efforts made to minimise and adjust for limitations. | 16a. Focus was on customizing to a specific setting.  16b. Not applicable. Potential influencing factors not measured.  16c. Discussion: planned discussions with client agency. |
| [17. Conclusion](https:/resources.equator-network.org/reporting-guidelines/squire/items/conclusion.html?utm_source=squire&utm_medium=checklist&utm_campaign=SQUIRE_2_0_2016_v1_1) | 17a. Usefulness of the work  17b. Sustainability  17c. Potential for spread to other contexts  17d. Implications for practice and for further study in the field  17e. Suggested next steps | 17a. Illustrative example that may provide readers suggestions for their own work.  17b. TBD. Long term impact of training and in-house capacity building for training not yet established.  17c. Unknown  17d and 3. Discussion and Appendix |
| **Other information** |  |  |
| [18. Funding](https:/resources.equator-network.org/reporting-guidelines/squire/items/funding.html?utm_source=squire&utm_medium=checklist&utm_campaign=SQUIRE_2_0_2016_v1_1) | Sources of funding that supported this work. Role, if any, of the funding organisation in the design, implementation, interpretation and reporting. | First page |

APPENDIX B: Final One-day Training Outline and Learning Objectives

| Time | TIDES**^TM^** Area of Focus |
| --- | --- |
| 8:30-9:00 am | Introductions |
| 9:00-10:00 am | Trauma-Informed Care to Prevention |
| 10:00-10:30 am | Reflection Activity |
| 10:30-1045 am | *Break* |
| 10:45 am -12:00 pm | De-escalation, Team Response, Code White Response |
| 12:00-12:15 pm | *Break* |
| 12:15-1:00 pm | Simulation |
| 1:00-2:00 pm | *Lunch* |
| 2:00-3:30 pm | Red Zone Management & Self-Protection Skills |
| 3:30-3:45 pm | Post Training Self Protection Skills – competency checks |
| 3:30-4:00 pm | Post incident debrief |
| 4:00-4:30 pm | Closing & post training evaluation |

| **Topic** | **Learning Objective** |
| --- | --- |
| **Culture of Safety** | - **Explore and define a safe culture as it relates to working with older adult populations** |
| **A Trauma-Informed Approach to Prevention** | - **Define Trauma** - **Understand how trauma affects older adults** - **Identify and utilize a trauma-informed approach to prevent and manage challenging behaviour** |
| **Collaborative De-escalation** | - **Identify strategies for collaborative de-escalation** - **Identify strategies for managing person reactions in the moment** |
| **Team Response to Escalated Situations and Code White Response** | - **Demonstrate understanding of staff roles and responsibilities during an escalated situation.** - **Apply team response protocols to code white situations** |
| **Trauma-aware self-protection skills** | - **Learn and understand the principles of trauma-aware self-protection** - **Practice the TIDES trauma-aware self-protection skills** |
| **Safe positioning when providing care** | - **Practice safe positioning when providing care** |
| **Post Event Staff Debriefing** | - **Discuss process of post event debriefing as it relates to psychological safety post-incident** |

APPENDIX C: Numeric and statistical details for Table 2 and Figures 1-5

**Selected Evaluation Results (Staff Surveys) for LTFC-identified Objectives**

**TABLE 2:** Reducing Responsive Behaviours: In the past month, how safe have you felt at work?

| RESPONSE (baseline only) | n | % |
| --- | --- | --- |
| No response | 8 | 7% |
| Somewhat unsafe safe to Not at all safe | 26 | 22% |
| ‘Neutral’ | 15 | 13% |
| Somewhat safe to Completely safe | 69 | 58% |
| TOTAL | 118 | 100% |

Increasing Resident Cooperation: How often do residents cooperate with mandatory care procedures without resistance?

| RESPONSE (baseline only) | n | % |
| --- | --- | --- |
| No response | 7 | 6% |
| Rarely/never | 7 | 6% |
| Sometimes | 67 | 57% |
| Always/often | 37 | 31% |
| TOTAL | 118 | 100% |

**Figure 1:** Decreasing Staff and Resident Injuries; Reducing the Frequency of Code Whites: Minimizing the Need for Police Intervention: How effective do you feel the training you had [previously/today] will be in preventing ....

| RESPONSE | Baseline | | Post-Training | |
| --- | --- | --- | --- | --- |
|  | mean | s.d. | Mean | s.d. |
| Staff injuries | 3.8 | 0.78 | 4.3 | 1.00 |
| Resident injuries | 3.9 | 0.77 | 4.2 | 0.96 |
| Incidents from becoming a code white | 3.8 | 0.78 | 4.4 | 0.80 |
| Resident escalations so that you can avoid having to call 911 | | | | |
| Resident escalations so that you can avoid having to call 911 | 3.8 | 0.76 | 4.3 | 0.96 |
| TOTAL | 118 | | | |

**Selected Evaluation Results (Staff Surveys) for TIDES^TM^ Trainer-identified Additional Topics**

**Figure 2:** Perceptions of Safety

| RESPONSE | Baseline | | Post-Training | |
| --- | --- | --- | --- | --- |
|  | n | % | n | % |
| No response | 0 | 0% | 11 | 9% |
| Very unsafe (1) | 9 | 8% | 0 | 0% |
| 2 | 1 | 1% | 0 | 0% |
| 3 | 2 | 2% | 0 | 0% |
| 4 | 6 | 5% | 0 | 0% |
| 5 | 20 | 17% | 4 | 3% |
| 6 | 26 | 22% | 4 | 3% |
| 7 | 18 | 15% | 18 | 15% |
| 8 | 18 | 15% | 26 | 22% |
| 9 | 7 | 6% | 12 | 10% |
| 10 | 8 | 7% | 32 | 27% |
| Very safe (11) | 3 | 3% | 11 | 9% |
| TOTAL | 118 | 100% | 118 | 100% |

**Figure 3:** Self-efficacy

Baseline: mean 53.4 (s.d. 12.7)

Post-training mean 72.3 (s.d., 31.8)

Sample size (denominator): 118

**Figure 4:** Confidence

| RESPONSE | Baseline | | Post-Training | |
| --- | --- | --- | --- | --- |
|  | n | % | n | % |
| No response | 3 | 3% | 14 | 12% |
| Not confident at all (1) | 1 | 1% | 0 | 0% |
| 2 | 1 | 1% | 0 | 0% |
| 3 | 3 | 3% | 0 | 0% |
| 4 | 2 | 2% | 0 | 0% |
| 5 | 10 | 8% | 0 | 0% |
| 6 | 18 | 15% | 2 | 2% |
| 7 | 24 | 20% | 13 | 11% |
| 8 | 27 | 23% | 27 | 23% |
| 9 | 12 | 10% | 20 | 17% |
| 10 | 8 | 7% | 28 | 24% |
| Extremely confident (11) | 9 | 8% | 14 | 12% |
| TOTAL | 118 | 100% | 118 | 100% |

**Figure 5:** Effectiveness of training (overall)

| RESPONSE | Baseline | | Post-Training | |
| --- | --- | --- | --- | --- |
|  | n | % | n | % |
| No response | 9 | 8% | 11 | 9% |
| Very ineffective/ineffective | 5 | 4% | 4 | 3% |
| ‘Neutral’ | 27 | 23% | 4 | 3% |
| Effective/Very effective | 77 | 65% | 99 | 83% |
| TOTAL | 118 | 100% | 118 | 98% (due to rounding error) |
